# Prevention of Anthracyclines and HER2 Inhibitor-Induced Cardiotoxicity: A Systematic Review and Meta-Analysis

**DOI:** 10.3390/cancers16132419

**Published:** 2024-06-30

**Authors:** Ioanna Myrto Sotiropoulou, Nikolaos Manetas-Stavrakakis, Christos Kourek, Andrew Xanthopoulos, Dimitrios Magouliotis, Grigorios Giamouzis, John Skoularigis, Alexandros Briasoulis

**Affiliations:** 1 Department of Clinical Therapeutics, Faculty of Medicine, Alexandra Hospital, National and Kapodistrian University of Athens, 11528 Athens, Greece; joannemirtoanastasiou@gmail.com (I.M.S.); nikolaos.manetas-stavrakakis@nhs.net (N.M.-S.); 2 Department of Cardiology, 417 Army Share Fund Hospital of Athens (NIMTS), 11521 Athens, Greece; chris.kourek.92@gmail.com; 3 Department of Cardiology, University Hospital of Larissa, 41334 Larissa, Greece; andrewvxanth@gmail.com (A.X.); grgiamouzis@gmail.com (G.G.); iskoular@gmail.com (J.S.); 4 Department of Cardiothoracic Surgery, University Hospital of Larissa, 41334 Larissa, Greece; dimitrios.magouliotis.18@alumni.ucl.ac.uk

**Keywords:** cardiotoxicity, prevention, anthracyclines, HER2 inhibitors

## Abstract

**Simple Summary:**

Evidence on cardiotoxicity prevention and treatment strategies in patients receiving anthracyclines or HER2 receptor inhibitors, vital treatments for breast cancer and hematologic malignancies, is quite a significant field of cardio-oncology. The present systematic review and meta-analysis investigated cardioprotective strategies in these patients and found that dexrazoxane, angiotensin-converting enzyme inhibitors, and β-blockers could decrease more than half the cardiotoxicity symptoms, cardiac abnormalities risk, and cardiovascular risk, indicating, thus, that cardioprotective medication is necessary for patients under chemotherapy.

**Abstract:**

Background: This meta-analysis and systematic review aim to consolidate evidence on cardiotoxicity prevention and treatment strategies in patients receiving anthracyclines or HER2 receptor inhibitors, vital treatments for breast cancer and hematologic malignancies. By synthesizing existing research, the goal is to provide impactful insights that enhance patient care and outcomes. Methods: Comprehensive research across PubMed, Scopus, EMBASE, and the Cochrane Central Register for Controlled Trials was conducted, selecting clinical trials focusing on cardioprotection in anthracyclines or HER2 inhibitor-treated individuals. Effect sizes were computed using OpenMeta (Analyst), with leave-out meta-analysis to assess potential small study effects. Meta-regression explored treatment duration and sample size effects. Evidence quality for primary outcomes was evaluated using ROB, Robins 2, and Newcastle-Ottawa tools. Results: Twenty -three studies involving a total of 14,652 patients (13,221 adults and 1431 kids) were included in the current systematic review and meta-analysis. The risk of bias and methodological quality of the included studies suggested good and moderate quality. Patients prescribed β-blockers demonstrated a 74% lower likelihood of exhibiting cardiotoxicity symptoms (OR 1.736). Similarly, the use of dexrazoxane was linked to a threefold decrease in cardiac abnormalities risk (OR 2.989), and ACE inhibitor administration showed half the risk compared with the control group (OR 1.956). Conclusions: Through this systematic review and meta-analysis, it was shown that there is a reduction in cardiotoxicity from either anthracyclines or HER2 inhibitors in patients receiving pharmacoprophylaxis.

## 1. Introduction

Anthracyclines are chemotherapy drugs used for solid tumors and hematological malignancies. They impede cancer cell growth by generating free radicals and inhibiting DNA processes such as replication and transcription [1,2]. In the meantime, HER2 inhibitors target aggressive cancers overexpressing HER2 [3,4,5,6]. They bind to HER2 proteins to disrupt signaling pathways, inhibit heterodimerization, and induce antibody-dependent cellular toxicity against HER2-positive cancer cells [3,4,6].

Both anthracyclines and HER2 inhibitors have been associated with common side effects, including nausea, vomiting, hair loss, fatigue, and myelosuppression [7]. Cardiotoxicity, as a major side effect of anthracyclines and HER2 inhibitors, manifests as arrhythmias, cardiac myocyte damage, and valve alterations [8,9]. Cardiac toxicity is usually assessed using echocardiography, ECG, and cardiac or inflammatory biomarkers such as Troponin I, BNP, and hs-CRP [10,11,12,13].

Anthracycline-induced cardiac damage may prove reversible with early diagnosis and prompt treatment. While the combination of ACEIs and β-blockers is widely utilized, its efficacy in specific clinical scenarios and long-term benefits remain contentious. In contrast, anti-HER2 agent-induced cardiotoxicity manifests solely during treatment, rendering troponin monitoring ineffective. Current recommendations primarily advocate for agent withdrawal or dosage adjustment. The 2023 ESC guidelines advise treating therapy-related cardiac dysfunction in moderate to severe asymptomatic and symptomatic cancer patients akin to the general heart failure population. For class I patients with mildly reduced or preserved EF, therapy includes fluid restriction alongside diuretics or SGLT-2 inhibitors, while class II patients are recommended ACEIs, ARBs, MRAs, and β-blockers for management.

Given these risks, monitoring cardiac function and employing cardioprotective measures, including treatment combination, managing cardiovascular risk factors such as hypertension, obesity, and diabetes, and utilizing pharmaceutical prophylaxis, are essential [14,15]. Specifically, dexrazoxane inhibits free radical formation and protects heart cells from oxidative stress, while β-blockers reduce cardiac workload and regulate rhythm [16,17,18]. In addition, ACE inhibitors and ARBs slow LVSD progression and prevent heart failure in high-risk patients [19]. Finally, statins potentially shield the heart by reducing oxidative stress and inflammation [20].

The aim of this study is to determine the efficacy of these cardioprotective factors in minimizing cardiotoxicity associated with anthracyclines and HER2 inhibitors.

## 2. Materials and Methods

The protocol of the current systematic review and meta-analysis was based on the Preferred Reporting Items for Systematic Reviews and Meta-Analysis (PRISMA) guidelines. Obtaining consent from the patients participating in the research, as well as submitting the protocol to the National Ethics Committee, was not required for this type of study.

### 2.1. Inclusion and Exclusion Criteria

Randomized controlled trials, cohort studies and case-control studies investigating the effect of cardioprotective drugs in patients treated with either anthracyclines or HER2 inhibitors for malignant neoplasms were selected. Systematic studies, meta-analyses, surveys, conference abstracts, and trials on animals or in vitro were excluded.

### 2.2. Data Sources and Search Strategy

To identify and select all the related studies, an extensive search of databases PubMed (1964–2022), Scopus (2004–2022), EMBASE (1980–2022), and Cochrane Central Register For Controlled Trials—CENTRAL (1999–2022) was conducted. The following algorithms were used consecutively with the keywords “Cardiotoxicity Prevention, Anthracyclines, HER2 inhibitors”.

- ((Prevention) OR (Prolepsis)) AND (Anthracycline) AND ((Cardiotoxicity) OR (Cardiopathy) OR (Heart disease) OR (Cardiac damage)).
- ((Prevention) OR (Prolepsis)) AND ((Her2 antagonist) OR (Her2 inhibitor) OR (Human epidermal growth factor receptor 2 inhibitor)) AND ((Cardiotoxicity) OR (Cardiopathy) OR (Heart disease) OR (Cardiac damage)).

### 2.3. Screening and Data Extraction

During the selection of the bibliography, filters were applied (studies written in the English language and trials conducted to humans only) that were intended to refine the incoming volume of information. In order to ensure complete impartiality, the entire list of available sources was uploaded to the Rayyan platform, where two blinded researchers (IMS and NMS) proceeded to sort the articles independently. The process consisted of three separate consecutive stages. Initially, duplicate articles were removed and then, were assessed based on their title and abstract content. Those that met the inclusion criteria were selected for evaluation in their full form. 

Any disagreement between the two investigators on the selection of articles and their evaluation of the presence of bias was resolved by consensus.

### 2.4. Assessment of Risk of Bias

Two researchers (IMS and NMS) participated in ensuring the quality of the selected literature. In more detail, the Cochrane ROB2 tool was selected for the set of randomized clinical trials, which is structured in such a way as to consist of a set of standardized questions that assess quality in the areas of design, execution and trial results [21]. Non-experimental studies were assessed with the Newcastle-Ottawa scale which focuses on the selection population, the comparability between the control and intervention groups, as well as the verification of the final results [22]. Finally, the non-randomized clinical studies were evaluated with the ROBINS-1 tool.

### 2.5. Statistical Analysis

In the interest of obtaining homogeneity, two main sets were created with adult and pediatric populations and were classified according to the intake of a cardioprotective agent as well as to the method of cardiotoxicity assessment. Data synthesis was conducted separately for the adult subgroups that received β-blockers, ACEIs, or dexrazoxane. For the studies that examined cardioprotective drugs other than the above three, due to a lack of sufficient data, a quantitative analysis could not be performed. However, a description of their results is provided.

The statistical program OpenMeta Analyst software version 10.10 was used to perform the meta-analysis. Following the completion of the analysis, a meta-analysis was conducted by sequentially removing each study (leaving out meta-analysis). In addition, a meta-regression for sample size and duration of treatment was performed, as there were corresponding data in the studies. Finally, to assess publication reliability, the funnel plot was created and complementary, the *p*-value was calculated to control Egger’s test.

## 3. Results

### 3.1. Study Selection

**Figure 1:**
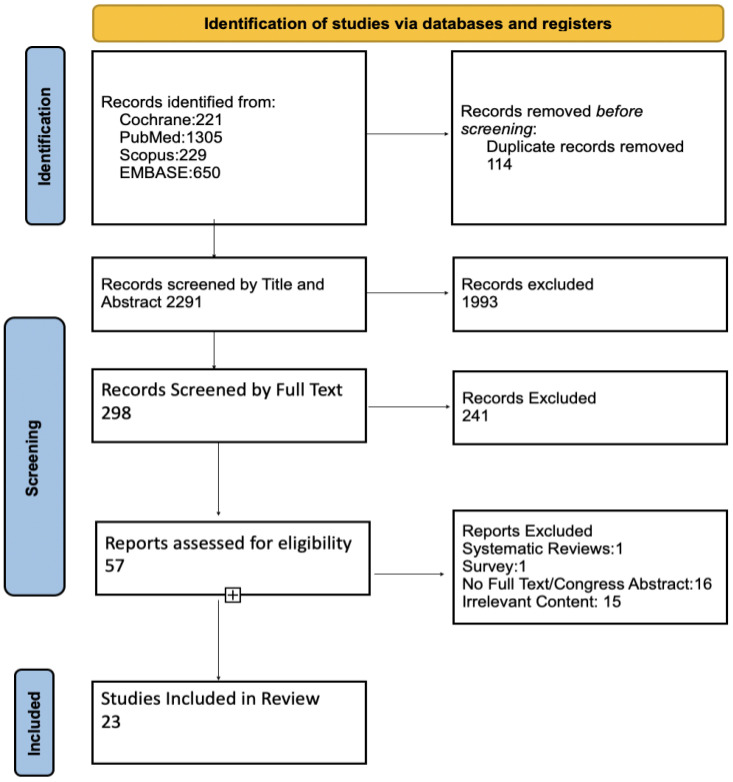
Flowchart of the systematic review and meta-analysis.

The flowchart illustrates the study selection process. A total of 2405 studies were identified, from which 57 were selected. From those, 34 studies were excluded at a later stage. Seventeen of them were excluded because their content was irrelevant to the researched topic. The remaining sixteen studies were excluded because they either did not provide enough data or because they were congress abstracts. At the end of the selection process, 23 studies were included in the analysis [16,17,20,23,24,25,26,27,28,29,30,31,32,33,34,35,36,37,38,39,40,41,42] (Figure 1).

### 3.2. Study Characteristics

A total of 23 studies comprising 14,652 subjects were included in the study, of which 13,221 were adults and 1431 were pediatric populations [16,17,20,23,24,25,26,27,28,29,30,31,32,33,34,35,36,37,38,39,40,41,42]. The adult group had HER(+) or HER(−) breast cancer as a primary malignant neoplasm, mainly stages II and III according to AJCC (American Joint Committee on Cancer), BMI in the overweight category, and preserved ejection fraction (defined as LVEF ≥ 50% in most studies). Subjects were scheduled to receive treatment with anthracyclines and HER2 inhibitors, and, in equal proportions, they had undergone radiation therapy. The cutoff for the systolic blood pressure and pulse at the beginning of each study protocol was 90 mmHg and 60 beats per minute, respectively. Possible pregnancy, existence of previous heart disease, and allergy to the intervention drug were the main reasons for exclusion. The pediatric group consisted of patients with hematological and neurological cancers [Acute Myeloid Leukemia (1103 patients) followed by neuroblastoma (95 patients) and Acute Lymphoblastic Leukemia (86 patients)] undergoing treatment with anthracyclines. Exclusion criteria were the presence of heart disease (including congenital anomalies) and Down’s syndrome. To evaluate cardiotoxicity between patients and controls, the most frequent measure was the comparison of LVEF (left ventricular ejection fraction) at the beginning and after the completion of treatment. In addition, the number of subjects with cardiotoxicity events, imaging findings in the cardiac ultrasound (strain, fractional shortening, e/e’, E/A, cardiac volume) and in the MUGA-scan and biomarkers (troponin, BNP) were measured.

### 3.3. Assessment of Risk of Bias and Applicability 

The majority of randomized clinical trials were of low risk for outcome data, three had a moderate risk in the randomization process, and only one was of high risk for the outcome measurement. Non-experimental studies scores varied between 7–8/9 of the total score in comparability and verification of results. Finally, the non-randomized clinical the non-randomized clinical studies were of low risk for bias (Table 1, Table 2 and Table 3). 

### 3.4. Systematic Review 

In this systematic review, several cardioprotective measures were examined across different studies. Candesartan was studied for cardioprotection in 332 patients across two trials [17,30]. Boekhout’s findings indicated a lower rate of LVEF reduction < 45% in patients receiving candesartan alongside anthracyclines, taxanes, and HER2 inhibitors compared with controls (11.3% vs. 14.4%) [30]. Similarly, Gulati’s study demonstrated a smaller change in LVEF at follow-up compared with baseline in the intervention group (−0.8, 95% CI −1.9 to 0.4 vs. −2.6, 95% CI −3.8 to −1.5) [17]. Assessment of cardiotoxicity, including several indices of echocardiography and biomarkers in clinical trials, is demonstrated in detail in Supplementary Table S1.

The efficacy of angiotensin-converting enzyme inhibitors (ACEIs) as prophylaxis during taxane chemotherapy or HER2 inhibitor treatment was assessed in six articles involving 7492 patients [29,31,32,33,34,41]. ACEI administration demonstrated a halved risk compared with controls (OR 1.956). However, Silber’s study found no significant association between ACEIs and stress levels in the left ventricle walls (LVESWS, *p* = 0.24) or percentage reduction in left ventricle size during systole (fractional shortening, *p* = 0.81) [41].

In addition, β-blockers were assessed across 10 articles involving 7891 patients [17,26,27,28,29,31,32,33,34,42]. A meta-analysis of patients evaluated for left ventricular ejection fraction changes before and after chemotherapy cycles revealed a 1.37 times lower likelihood of cardiotoxicity compared with controls. Wittayanukorn’s study, assessing β-agonists, was not included due to a different methodology (sum of ICD-9 codes concerning forms of cardiac disease), but indicated fewer cardiac events in the drug group over a 60-month follow-up [19.8 (95% CI 18.9–20.7) vs. 16.4 (95% CI 14.4–18.3)] [29]. Similarly, Wihandono’s study showed an improved left ventricular ejection fraction after six chemotherapy cycles in the intervention group (60.12 SD: 8.44 vs. 65.5 SD: 4.36) [32].

The use of statins involved 4673 patients across three articles [20,24,25]. Seicean’s study revealed a 2.3 times greater risk of LVEF < 55% in subjects not receiving statins (95% CI 1–5.1) [21]. Additionally, Arguelles found a lower LVEF in the control group at the treatment end compared with the intervention group, despite similar initial measurements (64.6%, 95% CI 62.2–67.1 *p* = 0.034 vs. 61.2%, 95% CI 59.6–62.8) [20]. Abdel’s study showed a nearly twofold increase in cumulative incidence among statin-naïve subjects (1.1% vs. 2.4%) [25].

Finally, the cardioprotective effect of dexrazoxane was investigated in seven studies involving 1891 patients (673 adults and 1218 children) [16,34,35,36,37,38,39,40]. In adult populations, cardiac events were three times more frequent in the control group compared to the dexrazoxane group. Venturini’s study further supported dexrazoxane’s benefits, showing a twofold decrease in left ventricular ejection fraction (LVEF ≤ 45%) compared with controls [36]. Similar results were observed in pediatric populations treated for blood neoplasms, with studies by Getz and Filomena demonstrating less decline in LVEF at the treatment end compared with controls [37,38]. Conversely, Elbl found comparable LVEF before and after cardiac stress in patients receiving anthracyclines (64 ± 3 and 62 ± 3 vs. 76 ± 3 and 76 ± 3 respectively) [40]. Choi’s study showed more intense early and final cardiotoxicity in the control group (38.1% vs. 25.5% and 14.3% vs. 2.2%) [39].

As a result of the large amount of information about chemotherapy side effects in the existing systematic review, it was possible to examine their prevalence. In particular, the predominant symptom was the existence of nausea and dizziness [27,31,34,35,36,37,42] while the next mentioned one was fatigue [31,34,36,37,42]. In addition, more than half showed a drop in hematological series (anemia, thrombocytopenia, leukopenia) [31,36,37,42] and inflammation of the vessels [17,31,37]. Finally, a number of patients presented disorders of either the skin (cheilitis, alopecia) [16,31,36,37,42] or the gastrointestinal system [16,31,36,37]. 

### 3.5. Meta-Analysis

#### 3.5.1. β-blockers

**Figure 2:**
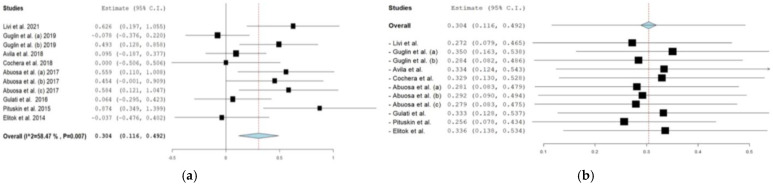
(**a**) Forest plot for measuring the efficacy of beta-blockers on LVEF [17,26,27,28,31,33,34,42]. Values greater than 0 indicate a positive effect of β-agonists on LVEF, while values smaller than 0 indicate a negative effect of β-agonists on LVEF. (**b**) The results of the “leave out meta-analysis” for measuring the effectiveness of beta-blockers on LVEF [17,26,27,28,31,33,34,42].

The analysis of β-blockers used for cardioprotection encompassed eight studies with 1107 patients [17,26,27,28,31,33,34,42]. Patients receiving β-blockers exhibit a 74% reduced likelihood of cardiotoxicity symptoms compared with controls (OR: 1.736), with a Cohen’s d index of 0.304 (95% CI = 0.116 to 0.492) (Figure 2a,b). Moderate heterogeneity (I2 = 58.47%, *p* = 0.007) is observed, and leave-out meta-analysis indicates no significant study effect. Larger sample sizes are associated with increased effect sizes (Cohen’s d −0.004, 95% CI = −0.007 to −0.001, *p* = 0.015), while treatment duration shows no significant association (Cohen’s d index −0.004, 95% CI = −0.027 to 0.019, *p* = 0.726) (Supplemental Figure S1).

#### 3.5.2. Dexrazoxane

**Figure 3:**
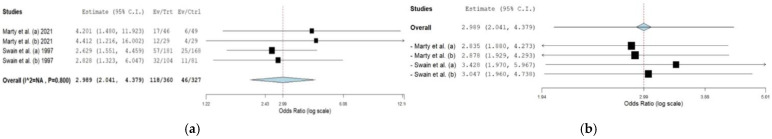
(**a**) Forest plot for measuring the efficacy of dexrazoxane on cardiac events [16,35]. The measure of the relationship calculated was the pooled odds ratio. Values greater than 1 indicate that cardiac events are more frequent in the control group than in the dexrazoxane group. (**b**) The results of the “leave out meta-analysis” for measuring the effectiveness of dexrazoxane in cardiac events [16,35].

The efficacy of dexrazoxane in preventing cardiotoxicity was assessed across four studies involving 687 patients [16,35]. A pooled odds ratio of 2.989 (95% CI = 2.041 to 4.379) indicated a threefold decrease in cardiac events compared with controls. Negligible heterogeneity (I2 = 0%, *p* = 0.80) is observed, with no significant study effect in the leave-out meta-analysis (Figure 3a,b). Increasing sample size shows no association with odds ratio (coefficient = −0.002, 95% CI = −0.005 to 0.002, *p* = 0.377), and treatment duration does not affect the pooled odds ratio (coefficient = −0.035, 95% CI = −0.104 to 0.034, *p* = 0.318) (Supplemental Figure S2).

#### 3.5.3. ACEs

**Figure 4:**
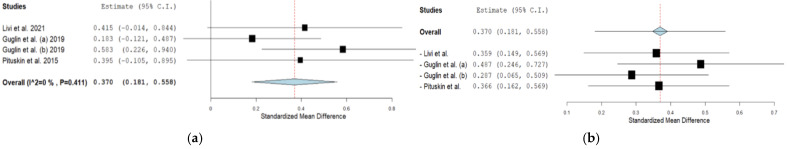
(**a**) Forest plot for measuring the effectiveness of ACEIs on LVEF [31,33,34]. (**b**) The results of the “leave out meta-analysis” for measuring the effectiveness of ACEIs on LVEF [31,33,34].

Angiotensin-converting enzyme inhibitors as a prophylactic measure during taxane chemotherapy or HER2 inhibitors were evaluated in five trials, including 440 patients [31,33,34]. ACE inhibitor administration shows half the risk compared with the control group (OR 1.956). Cohen’s d index is 0.370 (95% CI = 0.181 to 0.588), and nil heterogeneity (I2 = 0%, *p* = 0.411) is observed, with no significant study effect in the leave-out meta-analysis (Figure 4). Neither sample size nor treatment duration is associated with effect size, according to Cohen’s d index (−0.002, 95% CI = −0.007 to 0.002, *p* = 0.340 and −0.005, 95% CI = −0.043 to 0.033, *p* = 0.806) (Supplemental Figure S3).

#### 3.5.4. Funnel Plot

To estimate the publication error, the funnel plot was created, and the *p*-value for Egger’s test was calculated. β-blocker has a *p*-value of 0.048, indicating publication error, whereas Dexrazoxane and ACEIs demonstrate no risk for error (*p*-value 0.45 and 0.562, respectively) (Supplemental Figure S4).

## 4. Discussion

The findings of this current systematic review and meta-analysis support that the use of β-blockers and angiotensin-converting enzyme inhibitors is associated with a smaller decrease in LVEF in ultrasound measurements compared with the controls (OR 1.736 and 1.956, respectively) to a statistically significant degree. Patients who received dexrazoxane experienced three times fewer cardiac events during their course of treatment. Additionally, in studies where the drug of choice for cardioprotection was statins or an angiotensin II receptor blocker, the intervention group had better heart function at the end of treatment than the control group in parameters such as LVEF, cumulative incidence, and fractional shortening. It is important to mention that according to the meta-regressions conducted, the duration of treatment was not statistically significantly related to the range of results of each clinical trial. On the contrary, the size of the sample had a decisive role in the outcome of the result.

The findings of the present systematic review and meta-analysis align with previous meta-analyses and systematic reviews, indicating that pharmaceutical interventions such as β-blockers, ACE inhibitors (ACEIs), dexrazoxane, and statins exhibit potential in reducing the risk and severity of cardiotoxicity associated with anthracyclines or HER2 inhibitors [43,44,45,46,47]. Despite significant research efforts, consensus on the optimal approach to prevent anthracycline- or HER2 inhibitor-induced cardiac toxicity remains elusive. For instance, Gao et al.’s meta-analysis highlighted improved left ventricular ejection fraction (LVEF) at the end of treatment in patients receiving ACEI/ARB or β-blocker therapy, though no superiority among treatments was observed [47]. Conversely, another study suggested that β-blockers may attenuate LVEF reduction more effectively compared with ACEIs [48]. Furthermore, findings from the STOP-CA randomized clinical trial demonstrated a reduction in the number of patients experiencing a decline in LVEF greater than 10% with statin use as a cardioprotective measure [49].

In pediatric populations, research by Liesse et al. indicated a delay in cardiotoxicity among patients receiving dexrazoxane [50]. These findings collectively underscore the potential of various pharmaceutical agents in mitigating cardiotoxic effects associated with anthracyclines or HER2 inhibitors, providing valuable insights into tailored therapeutic strategies.

This study pioneers in comparison to the current bibliography for the inclusion of all the widely used methods of cardioprotection. Furthermore, having been designed and conducted according to the PRISMA guidelines, it had a clear and well-defined research objective. Multiple well-accredited databases were searched, and the resources were screened by two researchers blindly and independently. The majority of the studies included had a low risk for bias. The statistical analysis demonstrated a statistical significance in the measurements, whereas a leave-out meta-analysis and funnel plot to verify the results were applied. Additionally, the utilization of statistical analyses, including leave-out meta-analysis and funnel plot assessment, enhances the robustness of these findings. Finally, consideration of factors such as duration of treatment and sample size through meta-regressions adds depth to the interpretation of the results. Due to the great heterogeneity of the populations receiving the above leads, further clinical trials in specific subpopulations (age groups, common initial malignancy, same treatment time) were carried out so that there is a better assessment of the effectiveness of the pharmaceutical agents. Finally, it is important to evaluate the effect of the specific agents in patients with comorbidities as their action also manifests itself in other systems of the body that may affect the outcome of the treatment. The decision to use these drugs should be made on a case-by-case basis, taking into account each patient’s cardiovascular profile and the specific chemotherapy regimen being administered.

Nonetheless, this study has some limitations. To begin with, there was some heterogeneity according to the populations and methods of cardiotoxicity assessment making us unable to conduct a statistical analysis in all the papers included. Furthermore, the data sample consisted of women with breast cancer and pediatric populations with hematological malignancies, questioning the validity of generating its results in other populations or settings.

## 5. Conclusions

Through the current systematic review and meta-analysis, it was demonstrated a significant reduction in cardiotoxicity from either anthracyclines or HER2 inhibitors in patients receiving cardioprotective medication, including β-blockers, dexrazoxane and ACE inhibitors.

## Figures and Tables

**Table 1:** Newcastle-Ottawa quality assessment scale for case-control and cohort studies.

Case-Control	Selection				Comparability	Outcome			Total Score
Author	Case Definition Adequate	Representativeness of Cases	Selection of Controls	Definition of Controls	Of Cases and Controls Based on Design	Ascertainment of Exposure	Same Ascertainment	No Response Rate	
Arguelles et al., 2018 [20]	★	★		★	★ ★	★	★		7/9
**Cohort**	**Selection**				**Comparability**	**Outcome**			**Total Score**
**Author**	**Representativeness of exposed cohort**	**Selection of non** **-** **exposed**	**Ascertainment of exposure**	**Outcome not present at start of study**	**Of cohorts on basis of design**	**Asses** **s** **ment of o** **utcome**	**Folllow-up time enough for outcomes to occur**	**Adequacy of follow** **-** **up**	
Abdel et al., 2021 [25]	★	★	★	★	★★	★		★	8/9
Wittayanukorn et al., 2017 [29]		★		★	★★	★	★	★	7/9
Seicean et al., 2012 [24]		★	★	★	★ ★	★		★	7/9
Elbl et al., 2005 [40]		★	★	★	★ ★	★	★	★	8/9

★ indicates existence of the criterion.

**Table 2:** RoB2 tool for assessing risk of bias in randomized trials.

Author	Randomization Process	Deviations from Intended Interventions	Missing Outcome Data	Measurement of the Outcome	Result Selection
Livi et al., 2021 [31]	🟢	🟢	🟢	🟢	🟢
Wihandono et al., 2021 [32]	🟢	🟡	🟢	🟢	🟢
Guglin et al., 2019 [33]	🟢	🟢	🟢	🔴	🟢
Avila et al., 2018 [26]	🟢	🟡	🟢	🟢	🟢
Cochera et al., 2018 [27]	🟢	🟢	🟢	🟢	🟢
Abuosa et al., 2017 [28]	🟢	🟢	🟢	🟢	🟢
Boekhout et al., 2016 [30]	🟢	🟢	🟢	🟢	🟢
Gulati et al., 2016 [17]	🟢	🟢	🟢	🟢	🟢
Pituskin et al., 2015 [34]	🟢	🟢	🟢	🟢	🟡
Akpek et al., 2014 [23]	🟢	🟡	🟢	🟢	🟢
Elitok et al., 2014 [42]	🟡	🟢	🟢	🟢	🟢
Marty et al., 2005 [35]	🟡	🟢	🟢	🟢	🟢
Getz et al., 2020 [37]	🟡	🟢	🟢	🟢	🟢
Silber et al., 2004 [41]	🟢	🟢	🟢	🟢	🟢
Swain et al., 1997 [16]	🟢	🟢	🟢	🟢	🟢
Venturini et al., 1996 [36]	🟢	🟡	🟢	🟡	🟢

Green is low risk, yellow is some concern, and red is high risk in the RoB2 tool.

**Table 3:** ROBINS-1 tool for assessing risk of bias in non-randomized trials.

Author	Confounding	Selection	Classification Measurement of Exposure	Departures from Exposure	Missing Data	Measurement of Outcome	Reported Results
Filomena et al., 2019 [38]	🟠	🟡	🟡	🟡	🟡	🟡	🟡
Choi et al., 2010 [39]	🟢	🟡	🟡	🟡	🟢	🟡	🟡

Yellow is low risk, green is moderate risk, orange is serious risk, and red is critical risk in the ROBINS-1 tool.

## Data Availability

Data are available upon request.

## Supplementary Materials

The following supporting information can be downloaded at: https://www.mdpi.com/article/10.3390/cancers16132419/s1, Figure S1: Meta-regression for the effect of sample size (a) and treatment duration (b) on the efficacy of β-agonists on LVEF; Figure S2: Meta-regression for the effect of sample size (a) and treatment duration (b) on the efficacy of dexrazoxane on cardiac events; Figure S3: Meta-regression for the effect of sample size (a) and treatment duration (b) on the effectiveness of ACEIs on LVEF; Figure S4: Funnel plot of the meta-analysis measuring the effectiveness of β-blockers (a) or ACEIs (b) on LVEF and dexrazoxane on cardiac events (c). Table S1. Assessment of cardiotoxicity including several indices of echocardiography and biomarkers in clinical trials.
