# Supplementary material for: Prevention of Anthracyclines and HER2 Inhibitor-Induced Cardiotoxicity: A Systematic Review and Meta-Analysis
Source: Cancers (Basel). 2024 Jun 30;16(13):2419. doi: 10.3390/cancers16132419 (PMC11240691; doi:10.3390/cancers16132419)
Supplement: Supplementary file 1 [file cancers-16-02419-s001.zip › cancers-3064352-supplementary.pdf]

Supplementary Materials

Prevention of Anthracyclines and HER2 Inhibitor-Induced Cardiotoxicity: A Systematic Review and Meta-Analysis

Ioanna Myrto Sotiropoulou, Nikolaos Manetas-Stavrakakis, Christos Kourek, Andrew Xanthopoulos, Dimitrios Magouliotis, Grigorios Giamouzis, John Skoularigis and Alexandros Briasoulis

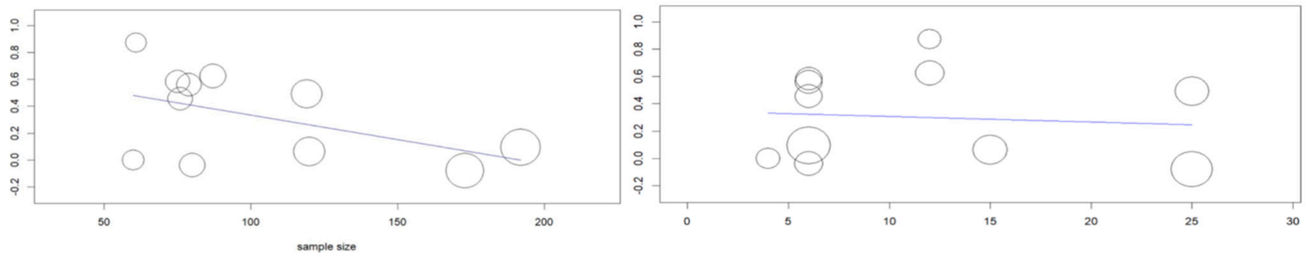

Figure S1. Meta-regression for the effect of sample size (a) and treatment duration (b) on the efficacy of β-agonists on LVEF.

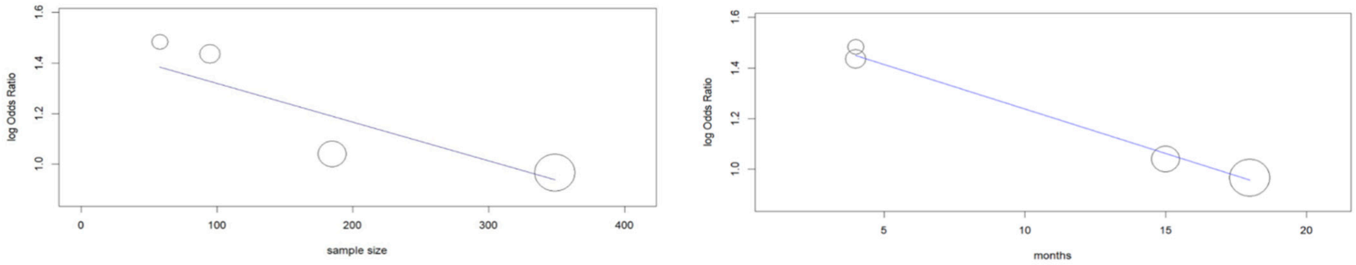

Figure S2. Meta-regression for the effect of sample size (a) and treatment duration (b) on the efficacy of dexrazoxane on cardiac events.

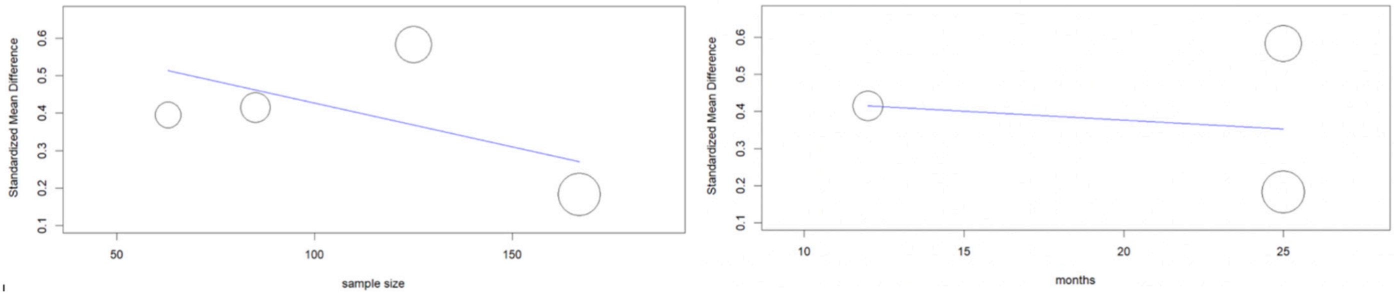

Figure S3. Meta-regression for the effect of sample size (a) and treatment duration (b) on the effectiveness of ACEIs on LVEF..

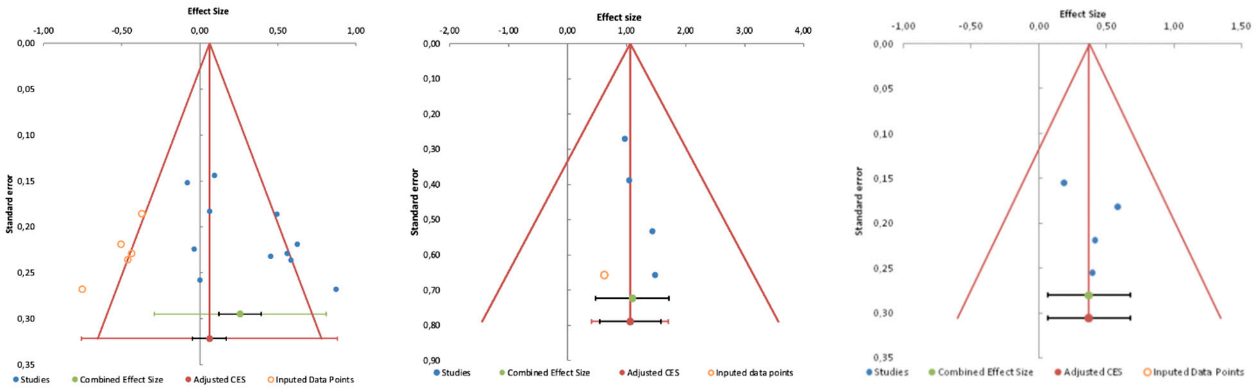

Figure S3. Funnel plot of the meta-analysis measuring the effectiveness of: β-blockers (a) or ACEIS (b) on LVEF and dexrazoxane on cardiac events (c).

Table S3. Assessment of cardiotoxicity including several indices of echocardiography and biomarkers in clinical trials.

**Author,Year**	**Extra Parameters Assessing Cardiotoxicity**
Swain et al 1997	Number of patients with CHF
Silber et al.,2001	• Fractional shortening<br>• Stress Velocity Index<br>• Global left ventricular end-systolic wall stress<br>• Maximal Cardiac Index
Elbl et al.,2005	• Fractional shortening<br>• Systolic Blood Pressure<br>• Diastolic Blood Pressure<br>• Heart Rate
Marty et al.,2005	• Incidence of Clinical Congestive Heart Failure<br>• Incidence of Cardiac Events
Akpek et al.,2014	• End-diastolic volume<br>• End-systolic volume<br>• Systolic Blood Pressure<br>• Diastolic Blood Pressure<br>• Heart Rate E/A and E/E'<br>• Mitral E, A<br>• Mitral deceleration time<br>• Lateral e'<br>• B-type natriuretic peptide<br>• Creatine kinase<br>• Troponin<br>• Oxidative biomarkers (Total Antioxidant Capacity, Total Organic Carbon)
Elitok et al.,2014	• Left atrial diameter<br>• Septal and Lateral Strain+rate+systolic peak<br>• Left ventricular systolic and diastolic size<br>• Fractional shortening<br>• Isovolumic relaxation time<br>• E/A, E, A
Pituskin et al.,2015	• End-diastolic volume<br>• End-systolic volume<br>• Systolic Blood Pressure<br>• Diastolic Blood Pressure<br>• Heart Rate

	• Left ventricular mass
Boekhout et al.,2016	• B-type natriuretic peptide<br>• New York Heart Association classification
Gulati et al 2016	• Right ventricular ejection fraction<br>• Left ventricular global longitudinal strain<br>• E/E', E/A, E'<br>• Left atrial end-systolic volume<br>• Troponin<br>• B-type natriuretic peptide
Abuosa et al., 2017	• End-diastolic volume<br>• End-systolic volume<br>• E/A and E/E'<br>• Systolic Blood Pressure<br>• Diastolic Blood Pressure<br>• Heart Rate<br>• Diastolic Time
Avila et al 2018	• Troponin<br>• B-type natriuretic peptide<br>• Number of patients with abnormal echo diastolic function
Cochera et al.,2018	• Global Longitudinal Strain + rate<br>• Circumferential Strain+rate<br>• Radial Strain+rate<br>• Tissue Doppler Flow Velocity septal<br>• End-diastolic volume<br>• End-systolic volume<br>• Shortening Fraction<br>• E/A, E/E'<br>• Mitral annular plane systolic excursion<br>• Isovolumic relaxation time
Filomena et al.,2019	• Left ventricular ejection fraction <50%<br>• End-diastolic volume<br>• End-systolic volume<br>• Global Longitudinal Strain<br>• Left ventricular mass<br>• Left atrial volume<br>• Right atrial volume<br>• A, E, E'<br>• Right ventricular diameter<br>• Stroke volume<br>• Pulmonary artery systolic pressure<br>• Inferior vena cava (mm)<br>• Pericardial effusion
Guglin et al.,2019	• Left ventricular ejection fraction drop by 10%<br>• Left ventricular ejection fraction drop to <50%<br>• End-diastolic volume<br>• End-systolic volume<br>• B-type natriuretic peptide
Getz et al.,2020	• Left Ventricular Systolic Dysfunction Grade 2 and 3<br>• Shortening Fraction
Livi et al., 2021	• Global Longidutinal Strain

	• End-diastolic volume<br>• End-systolic volume<br>• Left Atrial Volume<br>• Systolic Blood Pressure<br>• Diastolic Blood Pressure<br>• Heart Rate<br>• E/A and E/E'
